# The Influence of Plant Growth-Stimulating Bacteria on the Glutathione-S-Transferase Activity and the Toxic Effect of the Herbicide Metsulfuron-Methyl in Wheat and Canola Plants

**DOI:** 10.3390/toxics12120886

**Published:** 2024-12-05

**Authors:** Darya Chetverikova, Margarita Bakaeva, Sergey Starikov, Aliya Kendjieva, Sergey Chetverikov

**Affiliations:** Ufa Institute of Biology of Ufa Federal Research Centre of the Russian Academy of Sciences, 450054 Ufa, Russia; margo22@yandex.ru (M.B.); senik0406@gmail.com (S.S.); aliya_kendzieva@mail.ru (A.K.); che-kov@mail.ru (S.C.)

**Keywords:** acetolactate synthase, biostimulant, herbicide persistence, glutathione-S-transferase, metsulfuron-methyl, plant growth-promoting bacteria

## Abstract

The ability of some rhizosphere bacteria to mitigate herbicidal stress in cultivated plants may be useful in agriculture and bioremediation. There is poor understanding of how bacteria directly or through herbicide degradation affect the biochemical processes in plants exposed to sulfonylurea herbicides. In this study, treatment with a combination of herbicide metsulfuron-methyl (MSM) and bacteria (*Pseudomonas protegens* DA1.2 or *P. chlororaphis* 4CH) of wheat (*Triticum aestivum* L.) and canola (*Brassica napus* L.) plants was carried out. Activity of glutathione-S-transferase (GST), an important enzyme for the herbicide detoxification, and acetolactate synthase (ALS), a target for MSM in plants, was measured by spectrophotometric assays. MSM residues were analyzed using the HPLC-MS. Then, 24 h after bacterial treatment, GST activity increased by 75–91% in wheat and by 38–94% in canola. On the 30th day, a decrease in MSM in the soil associated with bacterial treatment was 54.6–79.7%. An increase in GST activity and acceleration of MSM degradation were accompanied by a decrease in inhibition of the ALS enzyme in plants, which indicated a mitigation of the toxic effect. The results obtained are evidence that rhizospheric bacteria can have beneficial effects on plants exposed to MSM due to the combination of abilities to directly affect detoxification enzymes in plants and degrade MSM in the soil.

## 1. Introduction

The active search for herbicide safeners began at the end of the 20th century. According to Jia et al. [1], more than 20 safeners had been commercialized as of 2022, and they are widely used in agricultural production. Of these, nine are designed to protect crops (mainly maize and rice) from the toxicity of sulfonylurea herbicides. Usually, safeners are chemical compounds of various groups: dichloroacetamides, carboxylic acid derivatives, oxime ethers, aromatic heterocycles, urea, thiocarbamate, amide, phytohormones, etc. [2,3,4]. Previous studies [4,5,6] indicate that safeners activate the metabolic pathways of herbicide detoxification in plants.

Metsulfuron-methyl (MSM) is a sulfonylurea herbicide that is widely used in modern agriculture due to its low cost, low doses, and effects on many weed species. The action of the herbicide is based on the inhibition of acetolactate synthase (ALS) enzyme, which is critically important for the synthesis of the amino acids valine, leucine, and isoleucine in plants [7]. The herbicidal effect of MSM is selective. It does little harm to wheat, barley, rye, oats, and pasture cereals due to their high ability to neutralize this compound [8]. However, it is toxic for dicotyledonous crops such as flax, beetroot, peas, soybeans, lentils, rapeseed, potatoes, and others. The residual amount of MSM usually does not exceed micrograms per gram of agricultural soil [9]. Nevertheless, it may cause damage to sensitive plant species in crop rotation systems at extremely low concentrations in the soil [10,11,12]. Finding simple and eco-friendly ways to protect sensitive cultures from MSM residues is important for agricultural practice and bioremediation. Modern ideas about sustainable development include the use of beneficial microorganisms to stimulate the growth of agricultural plants, protect them from diseases and pests, improve soil fertility, and remediate xenobiotic-contaminated soils. The ability of bacteria to mitigate herbicidal stress has attracted the attention of researchers relatively recently. For example, the native plant growth-promoting bacteria reduced the phytotoxicity of the herbicide imazethapyr for alfalfa (*Medicago sativa* L.) [13]. The results obtained by Jiang et al. [14] suggested that strain *Pseudomonas chlororaphis* PAS18 could alleviate the growth and physiological responses of *Pennisetum americanum* (L.) K.Schum caused by 20 mg∙kg^−1^ of atrazine. Bacterium *Pseudomonas putida* improved the physiological and biochemical parameters of durum wheat seedlings (*Triticum durum* Desf. Var. Mohamed Ben Bachir) and helped them to withstand the bleaching herbicide norflurazon [15]. The mitigation of herbicidal stress by strains *Pseudomonas protegens* DA1.2 [16], *Mesorhizobium* sp. MRC4 [17], and *Azotobacter salinestris* KF470807 [18] was also demonstrated. Mitigating herbicidal stress through microbial treatment has a number of potential benefits. Microbial preparations can have a prolonged effect if microorganisms survive in the soil or on the surface of plants. One microbial culture can have several useful qualities, help plants fight diseases, and improve the mineral nutrition of crops [19,20,21]. The search for effective microbial safeners is hindered by a weak understanding of the mechanisms of their protective action. There is some evidence that bacterial treatment affects the antioxidant system and reduced oxidative stress in plants [14,22].

A distinctive property of some microorganisms from chemical safeners is their potential of pesticide biodegradation in the soil. Basically, sulfonylurea herbicides are susceptible to chemical or microbiological degradation in the soil. The chemical decomposition of MSM depends on the pH of the soil [23] and is more typical for acidic soils. At a soil pH of 7.0 and above, MSM is relatively stable and persists longer in the soil. Its half-life increases to several months. Many species of soil bacteria and fungi are involved in the biological degradation of MSM [24,25,26,27]. The types of decomposition of sulfonylurea herbicides by microorganisms are also diverse. They can include oxidation, hydrolysis, and decarboxylation and differ significantly in different microorganisms [26]. Scientific articles [28,29,30] describe examples of the successful introduction of specially selected destructors into soils contaminated with sulfonylurea herbicides. The biological degradation of herbicides by such microorganisms is effective, safe for the environment, and considered as a promising alternative to physico-chemical methods of the remediation of contaminated soils. Therefore, the study of the contribution of microbial safeners to the degradation of sulfonylureas is of practical interest.

Plants metabolize herbicides using a three-stage detoxification system [31]. At the first stage, the herbicide molecule can be transformed by plant enzymes. At the second stage, these metabolites conjugate with sugars or the tripeptide glutathione. GSTs are a family of enzymes involved in this process. At the third stage, conjugates are recognized by ATP-binding transporters and transferred to a vacuole or apoplast. GSTs and cytochrome P450s are among the most important plant enzymes involved in metabolic resistance to sulfonylurea herbicides [31]. GSTs have attracted a lot of attention from researchers because of their role in herbicide selectivity and weed resistance to herbicides [32,33,34]. The relationship between herbicide resistance and GST activity has been confirmed by experiments on transgenic plants actively expressing the GST gene [35]. In cereals, much of the protective effect of chemical safeners has been attributed to increases in the detoxification capacity of plants. In particular, safeners enhance the expression of GSTs [36]. The treatment of *Arabidopsis* seedlings growing in a liquid medium with various chemical safeners similarly resulted in enhanced GST activities toward a range of xenobiotics [37]. The effect of microbial seiners on the activity of plant GSTs has not been widely studied.

Previously, we found that several strains of microorganisms isolated by us mitigate herbicidal and oxidative stresses in monocotyledonous and dicotyledonous plants [22]. The search for the properties of these bacteria that could potentially explain their protective effect is of interest.. This study investigated the impact of *Pseudomonas protegens* DA1.2 and *Pseudomonas chlororaphis* 4CH on glutathione-S-transferases (GST) activity in wheat and canola plants and MSM degradation in soil. It is known that GST activity was influenced by chemicals used as herbicide safeners [25], and GST activity can be quickly assessed by measuring the amount of glutathione conjugates. Therefore, the effect of rhizosphere bacteria on the enzymatic activity of GST was evaluated primarily before studying the gene expression of cytochrome P450s and other detoxification enzymes and transporters. The hypotheses of this work are as follows: (a) rhizospheric bacteria that mitigate the herbicidal stress of plants contribute to an increase in GST activity like chemical safeners; (b) the ability of bacteria to influence the degradation of MSM in the soil may explain their protection of plants from herbicides.

## 2. Materials and Methods

### 2.1. Experimental Details

The experiment was carried out in 2024 in the greenhouse of Ufa institute of biology of the Ufa Federal Research Centre of the Russian Academy of Sciences (location: Russian Federation, Ufa, latitude 54.44, longitude 55.58, altitude 158 m). The soil was taken from the arable layer of Chernozem Haplic (physical sand > 0.01 mm—61.4%, physical clay < 0.01 mm—38.6%, C_org_ 3.0%, N_tot_ 0.39%, P_Egner_ 145 mg/kg, K_Egner_ 117 mg/kg, pH_KCl_ 6.8). Before the sampling, the soil had not been used in agriculture for four years.

Herbicide MSM was provided by Euroagrocemicals LLC (Ufa, Russia). Seeds of common wheat (*Triticum aestivum* L. cv. Saratovskaya No 55), beet (*Beta vulgaris* L. cv. Cascade3 F1), and canola (*Brassica napus* L. cv. Kupol) were obtained from the Bashkir Research Institute of Agriculture of the Ufa Federal Research Centre of the Russian Academy of Sciences, Russia. Wheat and canola were chosen as research objects because they belong to different classes of plants (monocotyledons and dicotyledons) and differ significantly in sensitivity to MCM. They were used to study the effect of bacteria and their metabolites on the activity of GST and ALS. Beetroot was used as an MSM-sensitive plant only to test the phytotoxicity of the soil.

In order to study plant GST activity, wheat and canola plants were treated with MSM and bacteria. To do this, 5 germinated wheat or canola seeds were planted in each 0.5 L pot containing unpolluted soil. The pots were incubated in the greenhouse for 14 days. The photon flux density was 190 μmol∙m^−2^∙s^−1^, photoperiod was 14 h, temperature was 20–25 °C, and soil moisture was 60 ± 5% of the water-holding capacity. The plants were sprayed with MSM at a dose of 5 µg∙pot^−1^, bacteria at a dose of 10^5^ CFU∙pot^−1^, and a low-molecular-weight fraction of the culture liquid at a dose of 1 mL∙pot^−1^. Two negative controls were included in the experimental scheme: treated with herbicide (MSM) and untreated with herbicide (Control). Plants from the groups “Control” and “MSM” were treated with a diluted King B nutrient medium at a ratio of 1:10,000 (medium:water). All treatments are listed in Table 1. Plant samples were collected at 24 h and 72 h after treatment.

To investigate the degradation of MSM, the soil was intentionally polluted with MSM and incubated under controlled conditions. The soil was sifted through a 2 mm mesh, mixed with the MSM at a dose of 0.1 mg∙kg^−1^ of dry soil, divided into portions of 1 kg each, and placed in pots. Bacterial inoculum (10^5^ CFU∙mL^−1^) and dry soil were mixed in a ratio of 1:100 (m:m). Five wheat plants were planted in half of the pots. All treatments are listed in Table 2. They included two negative controls with and without plants in which soil was treated with MSM. The pots were incubated in the greenhouse. The photon flux density was 190 μmol∙m^−2^∙s^−1^, photoperiod was 14 h, temperature was 20–25 °C, and soil moisture was 60 ± 5% of the water-holding capacity. Soil samples were collected at 1, 7, 30, 60 days after application of herbicide. All the degradation tests were performed in a five-fold repetition.

### 2.2. Microorganisms and Their Cultivation

The DA1.2 strain of Pseudomonas protegens by Ramette et al. and the 4CH strain of Pseudomonas chlororaphis by Bergey et al. were isolated previously by the authors of the articles [38,39]. The bacterium DA1.2 was deposited in the All-Russian Collection of Microorganisms under number VCM B-3542D. These bacterial strains synthesize auxin and siderophores, fix atmospheric nitrogen, solubilize calcium-bound P (Table 3), and bio-control phytopathogenic fungi. Both bacteria retain their properties under herbicide MSM at a dose of up to 1 g per 1 kg of substrate. *P. chlororaphis* strain 4CHrif and *P. protegens* strain DA1.2rif were selected from populations of *P. chlororaphis* strain 4CH and *P. protegens* strain DA1.2 colonies that were resistant to 100 mg∙L^−1^ rifampicin when grown on King B agar amended with rifampicin.

The bacterial cultures were previously grown in King B medium (glycerol—10 g∙L^−1^, peptone—5 g∙L^−1^, K_2_HPO_4_—5 g∙L^−1^, MgSO_4_—1.5 g∙L^−1^) in an orbital shaker (160 rpm) at 28 °C for 72 h. They were then centrifuged. The microbial cells were washed with sterile water and used as an inoculum. A fraction containing metabolites with a molecular weight of less than 5 kDa was obtained from the supernatant by ultrafiltration through a filter «Vivaflow 50» (Sartorius, Germany).

For the monitoring, the introduced bacteria soils were serially diluted, and the appropriate dilutions were plated onto King B agar containing rifampicin.

To test bacterial strains for their ability to biodegrade MSM, they were cultured in different conditions: mineral M9 medium adding MSM as the sole carbon and energy source at a dose of 100 mg∙L^−1^; co-metabolic conditions using the mineral M9 medium and adding peptone, glucose, and MSM at doses of 500 mg∙L^−1^, 500 mg∙L^−1^, and 100 mg∙L^−1^, respectively. The nutrient media were buffered at pH 7, and a neutral pH was maintained in them during the experiment to avoid chemical degradation by acidic hydrolysis. In parallel, the bacteria were cultured in the same nutrient media without correcting the pH of the culture fluid. An aqueous suspension of bacteria at an amount of 10^5^ CFU per 100 mL of the medium in a 250 mL flask was added. Sterile media without inoculum served as a control. The flasks were placed in an orbital shaker (160 rpm) at 28 °C for 120 h. Each version of the experiment was conducted in twelve repetitions

### 2.3. Phytotoxicity Test

The phytotoxicity of the soil was checked using canola and beetroot as plants sensitive to the herbicidal action of MSM. Six pre-germinated seeds were planted in each 0.2 L pot and grown under artificial lighting. The photon flux density was 190 μmol∙m^−2^∙s^−1^, photoperiod was 14 h, temperature was 20–25 °C, and soil moisture was 60 ± 5% of the water-holding capacity. To test each treatment option, 10 pots of rapeseed and 10 pots of beetroot were used. The control plants were grown in soil uncontaminated with herbicide. Methsulfron-methyl has a weak effect on cotyledonous leaves and strongly inhibits the formation of the first leaves. Therefore, the plants from half of the pots were cut and weighed using analytical scales HR-250AZG (A&D Company, Limited, Tokyo, Japan) after 10 days, when 4 leaves had already formed. The average and standard error of 30 measurements were calculated. The remaining potted plants were used to analyze the ALS activity.

### 2.4. Determination of MSM Residues

Metsulfuron-methyl residues were analyzed using a modified method described in the article by Bossi et al. [40]. Soil samples were dried at room temperature in the dark and sifted through a 2 mm mesh. A soil sample weighing 20 g was mixed with 150 mL of 90% aqueous acetone, extracted for 15 min in an ultrasonic bath, and filtered. The extracts were evaporated to an aqueous residue (10–15 mL) on a rotary vacuum evaporator, and 90 mL of 0.1 M K_2_HPO_4_ was added. The aqueous residue was mixed with 20 mL of methylene chloride for 2 min, and the organic phase was ejected (three times). Then, the aqueous phase was acidified with 2M orthophosphoric acid to pH 3, and MSM was extracted with methylene chloride three times. The combined extract was filtered through a layer of anhydrous sodium sulfate (2 g) and evaporated on a rotary evaporator. To analyze the culture liquid, 100 mL of it was alkalized to pH 10 using 1 N NaOH, 25 mL of a mixture of hexane-methylene chloride (20:80, *v*:*v*) was added, and then the sample was prepared as described above.

The samples were analyzed using the HPLC system LC-20 Prominence with the diode matrix detector SPD-M20A (Shimadzu, Kyoto, Japan). The PerfectSil Target ODS-3 HD 5 µm (150 × 4.6 mm) column (MZ-Analysentechnik GmbH, Mainz, Germany) was used in gradient elution mode. The mobile phase A was acetonitrile. The mobile phase B was a 0.1% aqueous solution of acetic acid, the content of which varied in a range from 65% to 45% for 11 min. The flow rate was 0.5 mL∙min^−1^. The volume of the injected sample was 5 µL.

HPLC-MS analysis was performed on a liquid tandem chromatography-mass spectrometer LCMS-IT-TOF (Shimadzu, Kyoto, Japan) (at the AGIDEL UFSC RAS Equipment Collective Use Center). The device operated using electrospray ionization (ESI) in the positive ion mode with the following parameters: voltage of the high-voltage probe—4.5 kV; spray gas flow (N_2_)—1.5 L/min; temperature of the desolvation line (CDL) and the thermoblock—100 °C; pressure of the desiccant gas—100 kPa; cooling gas (argon)—105 kPa; voltage of the TOF detector—1.6 kV. The parent ions were recorded in the range of 100–1000 *m*/*z* with an accumulation time of 100 ms (event time—144 ms; repeat—1). MS2 ions were recorded in the range of 330–430 *m*/*z* under the following conditions: isolation of the precursor *m*/*z* 382.070, width—3 Da, ion accumulation time—100 ms, repeat—1. Trifluoroacetic acid solution was used as a standard sample for sensitivity and resolution settings, as well as for mass calibration (ion trap and time-of-flight analyzer). Software LCMS Solution 3.60 (Shimadzu, Kyoto, Japan) was used for data collection and processing.

Quantification of herbicide residues was carried out by comparing the peak area for the samples with the peak area of the analytical standards of MSM (ACCU standard, USA). For this purpose, standard samples of two types were prepared. Standard solutions containing 0.5, 1.0, 5.0, 10.0, and 20 µg mL^−1^ of MSM were injected into the HPLC to compare them with culture fluid extracts. Soil samples weighing 20 g were contaminated with 2 mL of standard solutions containing 0.01, 0.1, 0.2, 0.5, and 1.0 µg mL^−1^ methylfuron-methyl and analyzed in the same way as the experimental soils.

### 2.5. In Vivo Activity of Acetolactate Synthase

The in vivo assay ALS activity was represented by the accumulation of the substrate. It can monitor the activity inhibition by the ALS-inhibiting herbicide that is absorbed and even partially degraded by the plant. Simpson et al.’s [41] and Liu et al.’s [42] descriptions of the assay were used. In brief, the plants were sprayed with 0.5 M of 1,1-cyclopropanedicarboxylate (CPCA), a specific inhibitor of ketol-acid reductoisomerase (KARI) subsequent to ALS, to accumulate ALS enzyme substrate acetolactate. After 24 h, leaf samples were collected from the CPCA-treated plants, and 1 g of sample was extracted with 5 mL of 0.05 M sodium phosphate buffer (pH 7.2) and centrifuged for 10 min at 5000× *g*. Then, 0.5 mL supernatant was incubated at 60 °C for 10 min. Also, 0.1 mL of 1 M sulfuric acid was added to stop the catalysis of the ALS enzyme, and the incubation was maintained for 30 min to generate acetoin. Then, 1 mL of 0.5% (*w*/*v*) creatine (prepared in 2 M NaOH) and 1 mL of 5% (*w*/*v*) alpha naphthol (in 2 M NaOH) were added and kept at 37 °C for 30 min to transform acetoin into a red compound. The supernatant was obtained after an instant centrifugation and detected by using a spectrophotometer. The absorbance of the supernatant was measured at 530 nm (A530), and then the ALS activity was calculated as A530 per hour by 1 g fresh sample. Each sample had five replicates.

### 2.6. Glutathione-S-Transferase Activity

The leaves were cut from several plants and randomly divided into five samples of 1 g each. Frozen tissue was macerated on ice using a mortar and a pestle. Each powdered sample was thoroughly homogenized in 5 mL of sodium phosphate buffer (0.1 M, pH 6.5) that contained 20% glycerol, 14 mM dithiothreitol (DTT), and 1 mM ethylenediaminetetraacetic acid (EDTA) [43]. Homogenate was centrifuged at 9000× *g* (4 °C) for 20 min, and the supernatant was collected.

Glutathione-S-transferase activity was measured as described by Habig et al. [44]. The reaction mixture consisted of 50 mM potassium phosphate buffer (pH 6.5), aliquot of leaf extract (0.1 mL), 5 mM GSH, 0.4 mM 1-chloro-2,4-dinitrobenzene (CDNB), and 1% (*v*:*v*) ethanol in a final volume of 3.5 mL. Reactions were initiated with the addition of the CDNB substrate in ethanol. Enzymatic formation of 2,4-dinitrophenyl-S-glutathione was monitored for 5 min at 340 nm (E_340_ = 9.6 mM^−1^∙cm^−1^) using the spectrophotometer u-Violet DB SILab (Beijing Beifen-Ruili Analytical Instrument (Group) Co., Ltd., Beijing, China) and corrected for non-enzymatic controls. One unit of activity was defined as the amount of the enzyme that catalyzes the conversion of 1 μM CDNB per minute at 25 °C.

The measurements were carried out in five repetitions.

### 2.7. Statistical Analysis

The statistical analysis was performed using the software STATISTICA version 10 (TIBCO Software Inc., Palo Alto, CA, USA). The Shapiro–Wilk test was used to check the normality of the data. The significance of differences between the amount of MSM in the soil and nutrient medium, the number of bacteria, the GST activity, the weight of plants, and ALS activity in the phytotoxicity test was assessed using ANOVA with Duncan’s post hoc test for normally distributed data (*p* ≤ 0.05). A nonparametric Kruskal–Wallis test with Mann–Whitney U test (*p* ≤ 0.05) was used to process a dataset on ALS activity in an experiment in which GST activity was studied.

## 3. Results

### 3.1. GST Activity in Leaves Extracts of Bacteria-Treated Plants

**Figure 1:**
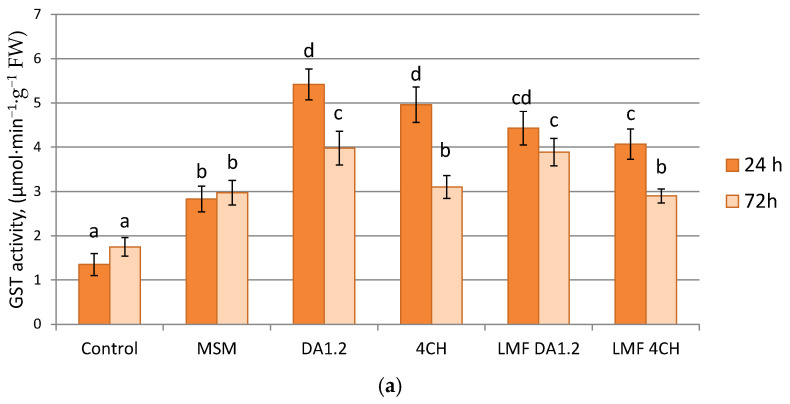

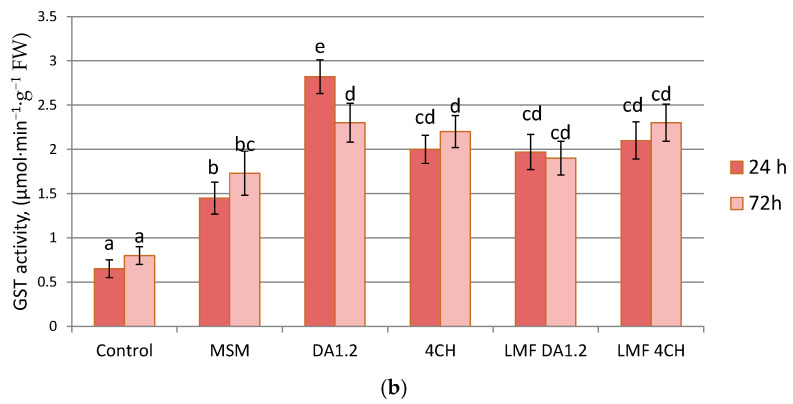
The effect of treatment with bacteria, a low-molecular-weight fraction (LMF) of their culture fluid, and metsulfuron-methyl (MSM) on glutathione-S-transferases (GST) activity in leaf extracts of wheat (**a**) and canola (**b**); enzyme activities are presented as average and standard errors (n = 5, Duncan’s test); significantly different means are indicated by different letters (*p* ≤ 0.05); control—herbicide and bacteria were not used, DA1.2–strain Pseudomonas protegens DA1.2, CH4—strain *P. chlororaphis* CH4.

Wheat and canola plants aged 14 days were sprayed with the herbicide MSM and bacterial cultures diluted in water. Then, 24 and 72 h after that, the leaves were cut off and GST activity was immediately measured (Figure 1). All types of treatment significantly influenced the enzymatic activity of leaf extracts. The effect was stronger after 24 h and weakened after 72 h. In the herbicide-treated samples, GST activity increased by 116–123% for canola and by 70–110% for wheat compared with the control plants. If MSM treatment was combined with bacterial cultures, after 24 h, the GST activity in the leaves of canola and wheat was higher by 38–94% and 75–91%, respectively, compared with using only the herbicide. Both plant species reacted more strongly to contact with the strain DA1.2. When the live bacteria were replaced with a low molecular weight fraction of the culture fluid, the differences between these variants were significant mainly after 24 h.

**Figure 2:**
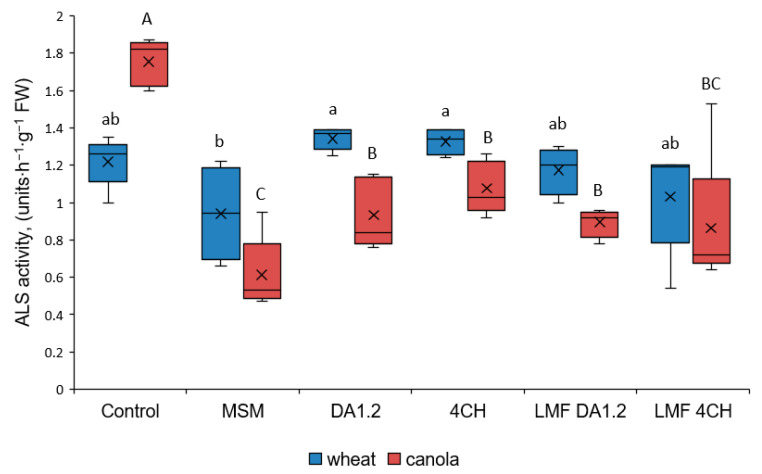
The effect of treatment with bacteria, a low-molecular-weight fraction (LMF) of their culture fluid, and metsulfuron-methyl (MSM) on acetolactate synthase (ALS) activity in leaves of wheat and canola; n = 5, U-test, significantly different (*p* ≤ 0.05) means within the “wheat” dataset and the “canola” dataset are indicated by different letters (lowercase and uppercase, respectively); control—herbicide and bacteria were not used, DA1.2 –strain *Pseudomonas protegens* DA1.2, CH4—strain *P. chlororaphis* CH4.

In parallel, some of the pots treated with methsulfuron-methyl and bacteria were used to measure the activity of ALS (Figure 2).

The toxicity of MSM is due to a decrease in the activity of this enzyme in plants. As expected, ALS activity in canola was more sensitive to the herbicide than ALS activity in wheat. In the leaves of canola plants sprayed with bacterial cultures and their LMF enzyme, ALS was more active than in leaves that were not exposed to them. A more noticeable increase (94.3%) in ALS activity was observed in canola plants after treatment with the strain 4CH. There was also a tendency to increase the activity of ALS in wheat leaves after treatment with the studied bacterial strains. However, the differences were not always significant.

### 3.2. Degradation of MSM in Soil

**Figure 3:**
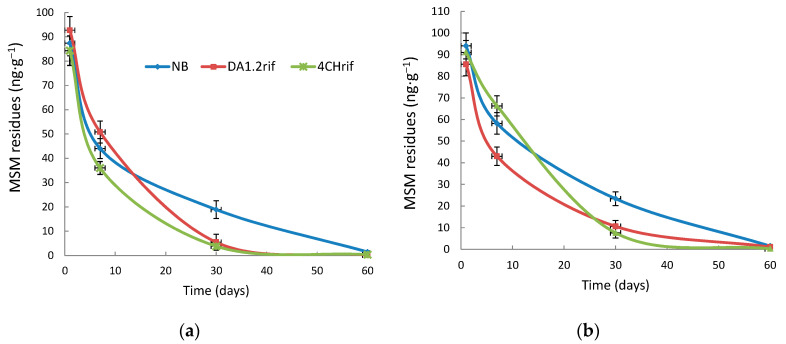
Degradation of MSM after introduction of strains *Pseudomonas protegens* DA1.2rif and *P. chlororaphis* 4CHrif in: (**a**) plant-free soils; (**b**) wheat-sown soils. NB—soil not treated with bacteria, DA1.2rif—soil treated with the rifampicin-resistant strain *Pseudomonas protegens* DA1.2rif, CH4—soil treated with the rifampicin-resistant strain *P. chlororaphis* CH4rif; data are presented as mean ± SE (n = 5, Duncan’s test, *p* ≤ 0.05).

In order to study the effect of growth-stimulating bacteria on the rate of MSM degradation, the soil was contaminated with 0.1 µg∙g^−1^ of MSM. The dynamics of MSM soil residues are shown in Figure 3. A fairly rapid degradation of herbicide was observed, which is associated with favorable soil temperature and moisture maintained in the laboratory. After 2 months of the experiment, less than 2% of the initial amount of MSM remained in the soil. For up to day 10, there were no significant differences in residual MSM between the treatment options. On day 30, samples exposed to bacteria significantly differed from the variants without their use. The decrease in the amount of MSM was 54.6–70.9% for strain DA1.2 and 67.4–79.7% for strain 4CH. On day 60, the samples with and without the bacterium 4CH were still different. But the quantity of herbicide was already low: 1.47–1.51 ng∙g^−1^ of soil without bacterial treatment and 0.30–0.39 ng∙g^−1^ of soil exposed to strain 4CH. In our experiment, wheat plants did not have a significant effect (*p* > 0.05) on the rate of MSM degradation in the soil.

Results of microbiological analysis of soils over the incubation period are shown in Table 4. The cells of the introduced bacterial strains survived in the soil during the entire observation period. They were multiplied in 10 days. Subsequently, their cell numbers decreased and approached the lower limit of the microbiological plating method for 60 days. There were no rifampicin-resistant bacteria detected in the control samples for any of the soil samples at any sampling time.

### 3.3. Degradation of MSM in Bacterial Cultures

Bacterial strains DA1.2 and 4CH were cultured under favorable conditions (temperature, aeration, and pH) in a liquid nutrient medium M9 supplemented with various carbon sources and MSM (Table 5). The strains DA1.2 and 4CH were not able to grow on MSM as the sole carbon or nitrogen source. Compared with the sterile environment, the decrease in MSM in the bacterial-inoculated medium was insignificant. After the addition of peptone and glucose to the nutrient medium, an active growth of bacteria was observed. If a neutral pH was maintained, it was accompanied by a slight increase in MCM removal from the medium. It was more noticeable in the case of using the strain 4CH. When the pH of the medium was not adjusted, glucose and peptone supported both the growth of bacteria and MSM degradation. Thus, the results imply that pH reduction and MSM removal with strains DA1.2 and 4CH from an enriched nutrient medium were apparently correlated.

### 3.4. Phytotoxicity of Soil Treated with Bacteria

The phytotoxicity of the soil was assessed 60 days after the introduction of bacteria into it by planting seedlings of MSM-sensitive crops in it. As presented in Table 6, herbicide-treated soil still inhibited the growth of canola and beet plants. Compared with the herbicide-free controls, the activity of ALS in their leaves was less by 76% and 80%, respectively. The result was a significant slowdown in plant growth and accumulation of shoot biomass.

The initial treatment of herbicide-containing soils with bacterial cultures had a beneficial effect on their phytotoxicity in the future. ALS activity in beet leaves was restored to 47–69% of the normal value of this indicator. ALS activity also reached a comparable level (44–77% of the control) in canola leaves. Various treatments affected the weight of the shoots. In the experimental variants where bacteria were used, canola and beet plants were larger. The weight of canola shoots increased by 67–133%, while the weight of beet shoots increased by 86–232% compared to those that were contaminated with methsulfuron-methyl alone. Significant differences in shoot weight and ALS activity were found between samples exposed to different bacterial strains. The 4CH strain was better suited to reduce soil toxicity caused by residues of the herbicide MSM. At the same time, a correlation was observed between the residual content of MSM, the weight of shoots, and ALS activity.

## 4. Discussion

According to the results described above, GST activity in wheat and canola leaf extracts reacted positively to treatment with bacterial strains *Pseudomonas protegens* DA1.2 and *P. chlororaphis* 4CH. Other authors also report an increase in the GST activity under the influence of *P. aeruginosa*, *Burkholderia gladioli* [45], and *Klebsiella pneumonia* [46]. Gaafar et al. [39] demonstrated the activation of the enzyme by cyanobacteria *Arthrospia platensis* and *Nostoc muscorum* lasting several days in bromoxynil-treated wheat. In this study, GST activity increased after treatment plants not only with a culture containing live bacterial cells but also with a low-molecular-weight fraction of the culture liquid. Presumably, some low-molecular-weight metabolites of bacteria performed the function of inducers. Among the previously identified substances produced by bacteria, auxin-like substances can claim this role. Bacteria *Pseudomonas protegens* DA1.2 can produce indolylacetic acid in a quantity of 870 µg∙L^−1^, and plant roots reacted to treatment with these bacteria as to treatment with auxins [16]. Auxins were the first phytohormones known to activate GST. IAA and the synthetic auxins 2,4-D and NAA have frequently been reported to induce the expression of various GST genes in different plant species [47]. Bočová et al. [48] reported that he short-term exposure of barley roots to IAA resulted in a significant increase in GST activity along the whole root tip of *Arabidopsis* seedlings, and the time-course spray applications of 1 mM 2,4-D caused an increase in the abundance of transcripts of GSTs from three classes [49]. In the study of Zhao et al. [50], some hormone-responsive and stress-responsive ones were predicted in the promoter of MdGSTs of an apple tree.

The obtained results indicated accelerated degradation of the herbicide MSM after soil treatment with bacterial cultures. So, the loss of herbicide for 30 days in soil samples treated with bacteria was 89.4–94.5%, whereas in untreated soils, it was 76.6–81.1%. In a similar pot experiment of Yang et al. [51], strain *Chenggangzhangella methanolivorans* CHL1, capable of efficiently degrading sulfonylurea herbicides, contributed to the removal of more than 91% of MSM and tribenuron-methyl after 7 days, significantly higher than the 25–36% degradation measured in non-inoculated treatments. The rate of chlorimuron-ethyl degradation by the bacterial consortium in the chlorimuron-ethyl-contaminated soil reached 80.02% at the end of a 60 d incubation period [29]. The inoculation with *Methylopila* sp. DKT in soil and peanut cultivation increased the bensulfuron-methyl degradation by 57.7% for 1 month [30], which suggests that both plants and the bacterial isolate play a key role in herbicide degradation. Compared with the 25.8% bensulfuron-methyl degradation efficiency in rhizosphere soil treated with 3.0 mg∙kg^−1^ without inoculation, 80.9% of bensulfuron-methyl was degraded 15 days post-inoculation with *Hansschlegelia zhihuaiae* S113 [52]. Thus, the effect on the degradation of the MSM of at least the bacterium *Pseudomonas chlororaphis* 4CH is comparable to the results of the other microorganisms proposed to increase the rate of degradation of sulfonylurea herbicides.

To explain the contribution of bacterial strains to the purification of the soil from MSM, the effect of their pure cultures on the herbicide added to culture media was studied. The biodegradation or biotransformation of sulfonilurea herbicides in pure cultures of microorganisms has been demonstrated many times [26]. According to the previous work, a wide range of microorganisms are involved in the process of degradation of sulfonylureas. Zanardini et al. [24] isolated *Pseudomonas fluorescens* strain B2 capable of co-metabolically degrading approximately 21% of the initially added 100 mg∙L^−1^ MSM within 2 weeks. More than 79% of MSM at concentrations of 0.10 mg∙L^−1^, 1.0 mg∙L^−1^, and 10.0 mg∙L^−1^ in pure culture was degraded by strain fungal strain MD after incubation for 7 days [25]. More than 97% of the initially added 50 mg∙L^−1^ MSM was depleted after 72 h when bacterial strain *Methylopila* sp. S113 utilized MSM as the sole carbon or nitrogen source [27]. Our bacterial strains were not able to use MSM as the only carbon source. However, if MSM was added to the microbial culture simultaneously with an organic substrate, then the degradation of the herbicide was comparable to data from other studies. Moreover, the herbicidal residues decreased faster if the pH of the nutrient medium was not regulated. This probably indicates the co-metabolism of MSM by the strain *P. chlororaphis* 4CH and the acceleration of chemical decomposition of the herbicide due to the acidification of the nutrient medium by both bacteria. The co-metabolism and acidohydrolysis of sulfonylureas provoked by microorganisms has also been recorded by other researchers. A co-metabolic degradation of tribenuron-methyl by bacterial strain *Pseudomonas* sp. NyZ42 isolated by Zhang et al. [53] from polluted agricultural soil was declared. The degradation efficiency of tribenuron-methyl was about 80% of the originally supplemented 200 mg∙L^−1^ tribenuron-methyl in a liquid minimal medium within four days, when either glucose or succinate was used as a supplemental carbon source. After the cultivation of *Aspergillus niger* in a rich medium for 28 days, the biodegradations were about 30% for chlorsulfuron and 33% for metsulfuron-methyl [54]. According to Wang [55], tribenuron-methyl degradation by strain *Serratia* sp. BW30 was dependent on glucose that was converted into lactic or oxalic acids. HPLC–MS analysis revealed two end-products from tribenuron-methyl degradation, and they were identical to the products from tribenuron-methyl acidohydrolysis. The soil is a mosaic of many microzones characterized by different physico-chemical conditions. Even if the introduction of bacteria does not affect the pH in the soil as a whole, microbiological acid production can reduce the pH in individual microzones at the boundary of soil particles and biofilms. For example, phosphorus is mobilized in this way [56,57]. The strains *Pseudomonas protegens* DA1.2 and *P. chlororaphis* 4CH are also capable of mobilizing phosphorus. We assume that, in the same way, they provoke acid hydrolysis of sulfonylureas. Another possible explanation for the accelerated degradation of MSM is the effect of introduced bacteria on the composition of the microbial community. However, additional research is required to verify this.

We tried to answer the question of whether differences in the amount of residual MSM or GST activity provoked by the introduction of bacteria lead to an improvement in the growth of test plants and the reaction of the target enzyme in them. It has been proven that the herbicidal effect of MSM is achieved by inhibiting the ALS enzyme. The resulting essential aliphatic amino acids and protein deficiency is accompanied by various secondary effects of ALS inhibition such as a depletion of intermediates of the pathway for some critical processes; a disruption of photosynthesis, transport and respiration system [58]; hindering the synthesis of DNA due to butanone accumulation; and often oxidative damage to biological molecules [59]. In relation to canola and beetroot sensitive to MSM, which we selected as test plants, the inhibition of the enzyme is clearly manifested. Against this background, the differences between the activity of the enzyme in plant leaves are clearly visible, depending on whether bacteria *P. protegens* DA1.2 or *P. chlororaphis* 4CH was previously introduced into the soil or not. Considering the biomass of the shoots, the phytotoxicity of the soil treated with bacteria was less. Similar trends in changes in the soil residues of MSM, soil phytotoxicity, and ALS activity with test plants allow us to conclude that the ability to biodegrade herbicides may be one of the aspects of the protective action of bacteria. However, the protective effect associated with biodegradation does not appear immediately after the introduction of bacteria but with a delay of a week or more.

To affect plants, bacteria must be in close contact with them. At the same time, it is desirable that the bacteria multiply and are not quickly displaced by the native microbiota. Then, the doses of safeners can be reduced and the beneficial effect will last longer. Therefore, the ability of bacteria to compete and successfully colonize the rhizosphere should be considered in the development and application of microbial safeners. The bacterial strains we tested demonstrated a good ability to colonize wheat roots. During the week, their number in the rhizosphere soil increased by several orders of magnitude. The rhizosphere is an ecosystem characterized by ecological richness [60,61]. There is a high competition for the exuded nutrients between the different organisms that inhabit it [62]. Apparently, there are properties of microorganisms that provide them with a competitive advantage in the rhizosphere, for example, it is the formation of biofilms, the ability to interact with plant immunity, the synthesis of antibiotics, etc. [63]. For the bacterial strains we tested, such properties include the synthesis of antimicrobial metabolites and siderophores, the mobilization of phosphorus from insoluble compounds, and nitrogenase activity. Bacteria also multiplied in the soil without plants, but their number was lower due to the lack of a rhizosphere effect.

## 5. Conclusions

Hence, it is possible that the ability of bacteria to serve as herbicide safeners is associated with a combination of several processes, including herbicide degradation and an increase in GST activity. Since the enzymes for the transformation of herbicides in plants react quickly to bacterial treatment, while the microbiological degradation of the herbicides in the soil takes time, the simultaneous manifestation of both properties may be important for the stable effect of microbial safeners.

GSTs are only a part of the mechanism of herbicide detoxification by plants, so it is worth investigating the effect of rhizosphere bacteria on oxidative enzymes and transporters as other parts of this mechanism. Also, field tests will be required to verify the effect of bacterial treatment on the persistence of sulfonylurea herbicides in soils, the activity of ALS, and herbicide detoxification enzymes in crops during the application of herbicides in crop rotation systems.

## Figures and Tables

**Table 1:** Treatment scheme 1.

Variant	Herbicide	Strain	Low-Molecular-Weight Fraction	Pots (0.5 L)
Control	− *	−	−	10
MSM	MSM	−	−	10
DA1.2	MSM	*P. protegens* DA1.2	−	10
4CH	MSM	*P. chlororaphis* 4CH	−	10
LMF DA1.2	MSM	−	LMF of *P. protegens* DA1.2	10
LMF 4CH	MSM	−	LMF of *P. chlororaphis* 4CH	10

* Not used.

**Table 2:** Treatment scheme 2.

Variant	Herbicide	Plant	Strain	Pots (1 L)
NB	MSM	− *	−	5
DA1.2 rif			rifampicin-resistant *Pseudomonas protegens* DA1.2rif	5
4CH rif			rifampicin-resistant *P. chlororaphis* 4CHrif	5
NB + W	MSM	wheat	−	5
DA1.2rif +W			rifampicin-resistant *P. protegens* DA1.2rif	5
4CHrif + W			rifampicin-resistant *P. chlororaphis* 4CHrif	5

* Not used.

**Table 3:** Properties of strains *Pseudomonas protegens* DA1.2 and *P. chlororaphis* 4CH [38,39].

Properties	Bacterial Strains	
	DA1.2	4CH
Indole-3-acetic acid production, mg∙L^−1^	0.870 ± 0.044	0.837 ± 0.040
Siderophores production (CAS agar), mm of zone	13 ± 4	8 ± 2
Calcium phosphate solubilization, mm of zone	21 ± 3	19 ± 3
Nitrogenase activity, nmol C_2_H_4_∙h^−1^∙mL^−1^	21.3 ± 3.6	30.5 ± 4.4

**Table 4:** The number of rifampicin-resistant bacteria in the soil after the introduction of strains *P. fluorescens* 4CHrif and *P. protegens* DA1.2rif, CFU∙g^−1^.

Treatments		Incubation Period, Day		
		10	30	60
Without plants	DA1.2 rif *	(7.2 ± 0.5)∙10^4^	(5.7 ± 0.3)∙10^4^	(1.5 ± 0.1)∙10^3^
	4CH rif	(2.8 ± 0.4)∙10^5^	(1.8 ± 0.2)∙10^4^	(3.0 ± 0.3)∙10^3^
Wheat plants	DA1.2 rif	(1.3 ± 0.2)∙10^5^	(6.0 ± 0.7)∙10^4^	(1.2 ± 0.1)∙10^3^
	4CH rif	(1.8 ± 0.2)∙10^5^	(6.8 ± 0.8)∙10^3^	(4.1 ± 0.5)∙10^3^
Wheat plants (rhizosphere)	DA1.2 rif	(4.9 ± 0.6)∙10^5^	(1.1 ± 0.2)∙10^5^	(7.7 ± 0.5)∙10^3^
	4CH rif	(3.8 ± 0.4)∙10^6^	(8.0 ± 0.3)∙10^5^	(5.3 ± 0.4)∙10^4^

* DA1.2rif—soil treated with the rifampicin-resistant strain *Pseudomonas protegens* DA1.2rif, CH4rif—soil treated with the rifampicin-resistant strain *P. chlororaphis* CH4rif; data are presented as mean ± SE (n = 12, Duncan’s test).

**Table 5:** The effect of nutrient sources on the bacterial growth and the degradation of MSM in the culture fluid of strains *P. fluorescens* 4CH and *P. protegens* DA1.2.

Bacterial Strain	Nutrient Medium	pH	MSM Removal, %	Cell Growth, CFU∙mL^−1^
DA1.2	M9	6.9	2.7 ± 0.9 ^a^**	(1.8 ± 0.3)∙10^5^
	M9 + glucose + peptone	7.2 *	8.4 ± 0.4 ^b^	(4.5 ± 0.4)∙10^8^
	M9 + glucose + peptone	6.2	96.3 ± 6.6 ^d^	(1.4 ± 0.2)∙10^8^
4CH	M9	6.8	3.5 ± 1.2 ^a^	(5.0 ± 0.6)∙10^4^
	M9 + glucose + peptone	7.0 *	35.3 ± 0.9 ^c^	(2.8 ± 0.2)∙10^8^
	M9 + glucose + peptone	5.8	97.6 ± 4.8 ^d^	(6.0 ± 0.5)∙10^8^

* Neutral pH was maintained during the experiment; ** data are presented as mean ± SE (n = 12, Duncan’s test); significantly different means are indicated by different letters (*p* ≤ 0.05).

**Table 6:** The effect of bacterial treatment on the toxicity of methsulfuron-methyl-polluted soil for test plants.

Treatments		Weight of Shoots, mg		ALS Activity, Units∙h^−1^∙g^−1^	
		Beet	Canola	Beet	Canola
Without plants	NB *	71 ± 9 ^a^**	240 ± 14 ^a^	0.34 ± 0.05 ^a^	0.54 ± 0.04 ^a^
	DA1.2 rif	236 ± 20 ^d^	462 ± 25 ^c^	1.17 ± 0.12 ^c^	1.56 ± 0.11 ^c^
	4CH rif	220 ± 16 ^d^	478 ± 24 ^c^	0.87 ± 0.07 ^bc^	1.48 ± 0.10 ^c^
Wheat plants	NB	92 ± 8 ^b^	221 ± 17 ^a^	0.40 ± 0.05 ^a^	0.57 ± 0.07 ^a^
	DA1.2 rif	172 ± 15 ^c^	370 ± 23 ^b^	0.79 ± 0.06 ^b^	0.98 ± 0.08 ^b^
	4CH rif	239 ± 17 ^d^	514 ± 18 ^cd^	0.99 ± 0.07 ^c^	1.72 ± 0.14 ^c^
Herbicide-free control		273 ± 21 ^f^	535 ± 27 ^d^	1.69 ± 0.14 ^d^	2.23 ± 0.21 ^d^

* NB—soil not treated with bacteria, DA1.2rif—soil treated with the rifampicin-resistant strain *Pseudomonas protegens* DA1.2rif, CH4—soil treated with the rifampicin-resistant strain *P. chlororaphis* CH4rif; ** data are presented as mean ± SE, n = 30 shoots for weighing, n = 5 samples for ALS assay; Duncan’s test, significantly different means in each column are indicated by different letters (*p* ≤ 0.05).

## Data Availability

The original contributions presented in this study are included in the article; further inquiries can be directed to the corresponding authors.
